# Modeling the theory of planned behavior to predict adults’ intentions to improve oral health behaviors

**DOI:** 10.1186/s12889-022-13796-4

**Published:** 2022-07-20

**Authors:** Mona Talal Rajeh

**Affiliations:** Department of Dental Public Health, Faculty of Dentistry, King Abdulaziz University, Jeddah, Saudi Arabia

**Keywords:** Adults, Intention, Oral health, Theory of planned behavior

## Abstract

**Background:**

The present study aimed to apply the theory of planned behavior (TPB) to identify predictors of adults’ intentions to improve oral health behaviors.

**Methods:**

This cross-sectional study was conducted with 1,328 adults living in the Jeddah city, Saudi Arabia. A 64-item questionnaire that evaluated behavioral intention, oral health knowledge (OHK) and TPB constructs (attitudes, perceived behavioral control, and subjective norms) was distributed. Descriptive statistics and structural equation modeling (SEM) were used to describe the data and examine the associations among the variables. A *p*-value of < 0.05 was considered significant.

**Results:**

The analysis revealed that the TPB model explained 72% of the variance in oral health behavioral intentions (OHBI), indicating a good model fit. The TPB constructs of attitudes (β = 0.299), subjective norms (β = 0.035), and perceived behavioral control (β = 0.144) were significant predictors of OHBI, whereas OHK was not. Attitude was the strongest predictor of intentions to improve oral health behaviors.

**Conclusions:**

The findings suggest that this model could be a helpful framework for designing oral health promotion and intervention programs. Such programs should focus on changing adults’ attitudes, positive influences from close relationships, and improving self-efficacy of OHB to improve their oral health behavior.

## Background

 Oral health contributes to the general health and overall well-being of an individual, and vice versa. Maintaining good oral health keeps teeth and gingiva healthy and improves individuals’ quality of life, bolstering their psychological and social well-being [1]. However, poor oral health, particularly periodontal diseases, has been associated with different health conditions, such as heart disease and diabetes [2–5].

Various measures to fight dental disease have been implemented and promoted, assisting many individuals worldwide [6]. Despite these efforts and significant improvements in oral health in different countries, oral health remains a public health concern [6]. According to the World Health Organization (WHO) and the Global Burden of Disease Study 2019, oral diseases affect 3.5 billion people worldwide [7]. Previous studies have shown that the prevalence of dental caries and periodontal diseases ranged from 50 to 80% in the Middle East, Latin America, and Asia [8, 9]. In Saudi Arabia, a recent systematic review reported the percentage of dental caries among adults’ population ranged from 50 to 90% [10]. In addition, several studies reported a high incidence and prevalence of periodontal disease among adults (20 years and above) living in different regions in Saudi Arabia [11, 12]. These studies revealed periodontal disease affect around 20 − 50% of Saudi adults’ population [11, 12]. These high burdens of dental disease emphasize the need to address this issue.

Accordingly, in 2020, the WHO and the World Dental Federation (Fédération Dentaire Internationale; FDI) proposed global goals and objectives as guidelines for policymakers to improve oral health and implement effective preventive programs in their countries [13]. For example, the WHO recommends practicing proper oral health behaviors such as reduced sugar intake, regularly brushing teeth twice a day, daily flossing, and regular dental checkups to prevent oral diseases such as dental caries [14, 15].

Many different theories have been proposed to explain human behavior; one commonly used in the oral health domain is the theory of planned behavior (TPB) [16]. Compared to other behavioral theories, the TPB is a flexible model that supports the inclusion of additional variables, increasing the proportion of variance explained and allowing for generalization to other contexts [17]. The TPB, developed by Ajzen [18], proposes that three constructs predict health-related behavioral intentions: attitudes, subjective norms, and perceived behavioral control. Attitude is defined as an individual’s feelings about the outcome of health behavior. Subjective norms are an individual’s beliefs about a given behavior, which can be influenced by close relationships. Perceived behavioral control is an individual’s perception of how difficult it is to carry out a particular oral health behavior [18]. Many researchers have successfully implemented the TPB to explain various health-related behaviors, such as smoking or healthy eating [19, 20]. Moreover, the TPB is a flexible model that can be modified to include additional variables [21]. Many studies have focused on extending the TPB, incorporating variables such as self-efficacy and knowledge [17, 22]. For example, Omondi et al. proposed a modified TPB model that included perceived knowledge as a predictor of intention toward physical activity and adhering to dietary guidelines in diabetic patients [23]. In dentistry, the theory has been applied to many oral health studies [23–27]. In 2020, Elyasi M et al. expanded the TPB to include the concept of a sense of coherence, which predicted parental adherence to preschoolers’ preventive dental visits [28]. In addition, the constructs in the TPB model are well-documented and strong predictors of oral health behavioral intentions (OHBI) in populations from various countries, such as Canada, Ireland, Romania, and Iran [24, 29, 30]. Previous studies have found that attitudes and perceived behavioral control strongly predict oral health intentions and/or behavior.

**Fig. 1:**
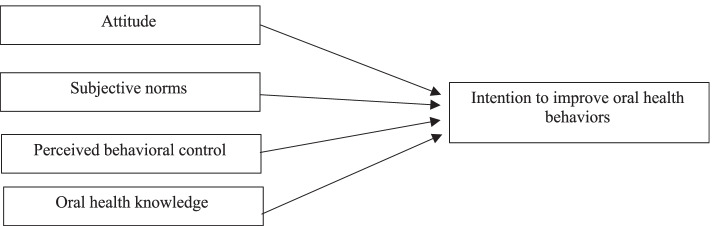
Proposed predictors of intentions to improve oral health behaviors

However, no studies have examined the application of TPB to predict OHBI in Saudi Arabia. Given the high prevalence of oral diseases among Saudi adults too, this study aimed to predict adults’ intentions to improve oral health behaviors by examining the influence of attitudes, subjective norms, perceived behavioral control, and OHK in Saudi Arabia (Fig. 1).

## Methods

### Study design and data collection

A cross-sectional descriptive study was conducted between August 2021- December 2021 among adults 20 years old and older, living in Jeddah city, Saudi Arabia and of any nationality. A structured online questionnaire was created using SurveyMonkey (San Mateo, California, USA) and advertised to the public through social media platforms such as WhatsApp, Facebook and Twitter. This study used a snowball sampling method, asking the participants to share the survey with eligible relatives and friends. The questionnaire’s cover page explained the study’s objectives and the contact information of the principal investigator. The first question asked was: “Are you 20 years old or older who live in the city of Jeddah and would like to participate?” By answering this question, ineligible participants were excluded. Paper questionnaires were also distributed at local malls to achieve higher response rates and to reach participants who had no access to social media. There were four sections of Jeddah city: north, south, east, and west. The largest shopping centers in each area were selected. The paper questionnaires were distributed at four shopping malls at the site. Data collectors settled in one area at the malls and encouraged all adults to participate in the study. There were two weeks scheduled for each mall from 5 p.m. to 9 p.m. Power analysis indicated that the minimum sample size for the structural equation model (power = 0.8, five latent variables, 40 observed variables, and an alpha level of 0.05) was 700 participants. Informed consent was obtained from all subjects and/or their legal guardian(s).

### Questionnaire

The questionnaire was adapted from the previous studies that demonstrated the validity and reliability of the study questionnaire [24]. The questionnaire was translated into Arabic following the forward-backward translation method. The questionnaire consisted of 64 closed-ended questions that examined the effect of the TPB constructs (attitudes, perceived behavioral control, subjective norms) on participants’ intentions to improve oral health behaviors. The questionnaire was divided into the seven following sections:

- Part 1: Demographic data (e.g., sex, age, marital status, and level of education).
- Part 2: Current OHB (e.g., frequency of brushing teeth, frequency of flossing, frequency of mouth wash use, date of the last dental visit and reason for the last dental visit). Each item was given a score weight, and the total values of the items were calculated. The summary oral hygiene behavior score ranged from 5 to 25. High scores indicated a high level of oral hygiene behavior (e.g., Higher frequency of dental visits reflects higher level of oral behavioral intention).
- Part 3: Oral health behavioral intentions (OHBI). This section included 5 questions that assessed how likely the participants were to engage in specific oral health behaviors (e.g., “I will brush my teeth more than twice a day”). Answers were provided on a 5-point Likert scale ranging from (1) extremely unlikely to (5) extremely likely.
- Part 4: Attitude (A). This section contained 5 items that asked the participants how they felt about performing certain oral health behaviors (e.g., “I feel regular tooth brushing with toothpaste twice a day is”). Participants responded on a 5-point Likert scale ranging from (1) extremely unpleasant to (5) extremely pleasant. A higher score indicated a more positive attitude toward OHBI.
- Part 5: Subjective norms (SN). In this section, two items for each behavior were used to assess subjective norms (e.g., brushing teeth: “People important to me would like me to regularly brush my teeth with toothpaste twice a day” and “People who influence me prefer that I regularly brush my teeth with toothpaste twice a day”). Participants provided their answers on a 5-point Likert scale ranging from (1) strongly disagree to (5) strongly agree.
- Part 6: Perceived behavioral control (PBC). In this section, three items were used to assess perceived behavior control for each behavior (e.g., flossing: “I find it easy to floss my teeth every day”, “I am able to floss my teeth every day” and “If I wanted to, I could floss my teeth every day”. Participants provided answers on a 5-point Likert scale ranging from (1) strongly disagree to (5) strongly agree. Higher score indicated higher PBC toward OHBI.
- Part 7: Oral health knowledge (OHK). This section was adopted from a previous study performed by Buunk-Werkhoven [27]. It consisted of 11 true/false questions that assessed knowledge of oral health behaviors, such as “If your gingiva does not bleed while brushing your teeth, there is nothing wrong with them” and “For tooth health, it doesn’t matter if you use the same toothbrush for a long time”. Items were scored as correct = 1 or incorrect = 0, and the total score was calculated by summing the 11 items; thus, the total OHK score ranged from 0 to 11. Higher scores indicated higher level of OHK.

### Reliability and validity

Three experts were consulted to ensure the clarity and content validity of the questionnaire. These experts evaluated the questionnaire items for clarity, importance, and relevance. Based on their comments and feedback, minor word choice and sentence structure changes were implemented to improve the clarity of the questionnaire. Next, the revised questionnaire was pilot tested on a convenient and representative sample of 30 adults who were not part of the study population to evaluate its construct validity and reliability. After the pilot test, a few additional modifications were made, including changes to clarify the sentence structure. Cronbach’s alpha was used to determine the reliability for each construct, and it is reported to be 0.67 for intention, 0.89 for attitude, 0.88 for subjective norms, 0.90 for perceived behavioral control and 0.97 for oral health knowledge.

### Statistical analysis

Descriptive statistics were provided via the statistical software STATA version Stata 23 (StataCorp, LLC., College Station, TX, USA). The normality of data distribution was assessed with the Kolmogorov-Smirnov test. A Spearman correlation was conducted to determine the strength of association between the measured variables and to estimate the inter-item correlation. The study hypotheses were then collectively tested using structural equation modeling (SEM), performed in IBM SPSS Amos 24 version 4.0. The SEM analysis was performed with all 4 variables from the multivariate model and value of chi-square obtained was greater the 0.05 which indicate a good fit; goodness of fit index (GFI), Root mean square error (RMSE), and Tuker Lewis index (TLI) showed the acceptable ranges overall. Smaller values of RMSE indicated the acceptability of the model, TLI and GFI ranges from 0 to 1 and values close to 1 showed the better fit of the model.

## Results

### Sample characteristics

The total number of questionnaires received was 1,330. Only two questionnaires were omitted because of missing data yielding 1328 completed questionnaires. Of the participants, most were female (68.6%), 20–30 years old (61.8%), Saudi nationals (85.8%), single (59.9%), and had a bachelor’s degree (53.8%). The majority of participants were employed (65.0%), worked in the private sector (62.3%), had monthly income < 10,000 SAR (72.0%), and did not smoke (65.0%) (Table 1).

**Table 1:** Demographic characteristics of participants

Variables	Description	n (%)
Sex	Male	417 (31.4)
	Female	911 (68.6)
Age	20–30	822 (61.8)
	31–40	328 (24.7)
	41–50	105 (7.9)
	> 50	75 (5.6)
Nationality	Saudi	1139 (85.8)
	Non-Saudi	189 (14.2)
Marital status	Single	748 (59.9)
	Married	380 (30.4)
	Divorced	95 (7.6)
	Prefer not to say	25 (2.0)
Level of education	High school or less	455 (34.2)
	Bachelor’s degree	716 (53.8)
	Master’s degree	125 (9.4)
	PhD	34 (2.6)
Occupation	Student	224 (19.9)
	Employed	732 (65.0)
	Unemployed	171 (15.2)
Working sector	Private sector	828 (62.3)
	Government sector	181 (13.6)
	Unemployed	319 (24.0)
Monthly income	< 10,000 SAR	956 (72.0)
	10,000–20,000 SAR	243 (18.3)
	> 20,000 SAR	129 (9.7)
Smoking status	Yes	465 (35.0)
	No	865 (65.0)

### Oral health practice and knowledge

Most of the respondents reported brushing their teeth twice a day (61.0%) and visiting a dentist for treatment or pain (53.5%) (Table 2). The mean (SD) OHK score of the respondents was 8.1 (1.7), and it ranged from 0 to 11 (Table 3). Female participants were more likely to state that gingival inflammation cannot disappear by itself (*p* < 0.000).

**Table 2:** Current oral health behavior

Variables	Description	n (%)
Tooth brushing frequency	Less than once a day	56 (4.2)
	Once a day	257 (19.3)
	Twice a day	811 (61.0)
	More than twice a day	206 (15.5)
Flossing frequency	Every day	288 (21.7)
	More than once a week	186 (14.0)
	Once a week	191 (14.4)
	Once a month	106 (8.0)
	Never	559 (42.0)
Mouth washing frequency	Every day	422 (31.8)
	More than once a week	201 (15.1)
	Once a week	185 (13.9)
	Once a month	92 (6.9)
	Never	429 (32.3)
Last dental visit	More than 2 years ago	217 (18.4)
	1–2 years ago	151 (12.8)
	6–12 months ago	298 (25.2)
	Less than 6 months ago	246 (20.8)
	Last month	270 (22.8)
Reason for dental visit	Check-up, tooth cleaning or scaling	525 (39.5)
	Treatment or pain	712 (53.5)
	No prior dental visits	93 (7.0)

**Table 3:** Oral health knowledge by sex

Question	Male N (%)		Female N (%)	
	Correct	Incorrect	Correct	Incorrect
For tooth health, it matters how often you eat sugary foods (like candy)	376 (90.2)	41 (9.8)	808 (88.7)	103 (11.3)
To prevent dental caries, it is insufficient to only brush the crown cover	359 (86.1)	58 (13.9)	795 (87.3)	116 (12.7)
When brushing your teeth, it is important to put little pressure on the toothbrush	249 (59.7)	168 (40.3)	505 (55.4)	406 (44.6)
To prevent dental caries, you should brush your teeth at least twice a day	334 (80.1)	83 (19.9)	715 (78.5)	196 (21.5)
For tooth health, it doesn’t matter if you use the same toothbrush for a long time	245 (58.8)	172 (41.2)	548 (60.2)	363 (39.8)
Gum inflammation can disappear by itself	279 (66.9)	138 (33.1)	697 (76.5)	214 (23.5)
Gum bleeding is a sign of gum disease	297 (71.2)	120 (28.8)	624 (68.5)	287 (31.5)
To prevent gum inflammation, you also have to clean between your teeth	355 (85.1)	62 (14.9)	798 (87.6)	113 (12.4)
Bad breath can be caused by gum disease	359(86.1)	58 (13.9)	806 (88.5)	105 (11.5)
Brushing your teeth before breakfast and before going to bed will enhance its preventive efficacy	388(93)	29 (7)	852(93.5)	59 (6.5)
If your gum does not bleed while brushing your teeth, there is nothing wrong with them	111(26.6)	306 (73.4)	203 (22.3)	708 (77.7)

### Theory of planned behavior variables

The mean score of respondents on all TPB variables was above average (neutral). Additionally, female participants had higher mean scores on all TPB variables (*p* < 0.001) except for OHK (Table 4).

**Table 4:** Descriptive statistics for variables in the model that could predict improvement in oral health behaviors by sex

	Male		Female		Total		*P*-value
	Mean	SD	Mean	SD	Mean	SD	*P* < 0.001
Intention	18.1	4.3	19.4	4.2	19.0	4.3	*P* < 0.001
Attitude	19.4	4.6	20.6	4.1	20.2	4.3	*P* < 0.001
Subjective norms	32.9	9.8	34.9	10.1	34.3	10.0	*P* < 0.001
Perceived behavioral control	57.8	57.8	61.1	11.2	60.1	11.7	*P* < 0.001
Oral health knowledge	8.0	1.8	8.1	1.6	8.1	1.7	*P* = 0.67

### Correlation among the variables

Correlation analysis was performed among the TPB variables, OHK and OHB. Intention was significantly correlated with attitudes, subjective norms, perceived behavioral control, oral health knowledge and current oral health behavior. However, oral health knowledge was not correlated with attitudes, subjective norms and current oral health behaviors (Table 5).

**Table 5:** Spearman’s correlations among the TPB variables, OHK, and OHB

	I	A	SN	PBC	OHK	OHB
Intention (I)	1					
Attitude (A)	0.435^a^	1				
Subjective norms (SN)	0.359^a^	0.310^a^	1			
Perceived behavioral control (PBC)	0.523^a^	0.545^a^	0.480^a^	1		
Oral health knowledge (OHK)	0.068^b^	− 0.034	0.038	0.135^a^	1	
Current oral health behavior (OHB)	0.519^b^	0.243^a^	0.279^a^	0.316^a^	− 0.007	1

^a^ Correlation was significant at the 0.01 level

^b^ Correlation was significant at the 0.05 level

### Structural equation model

**Fig. 2:**
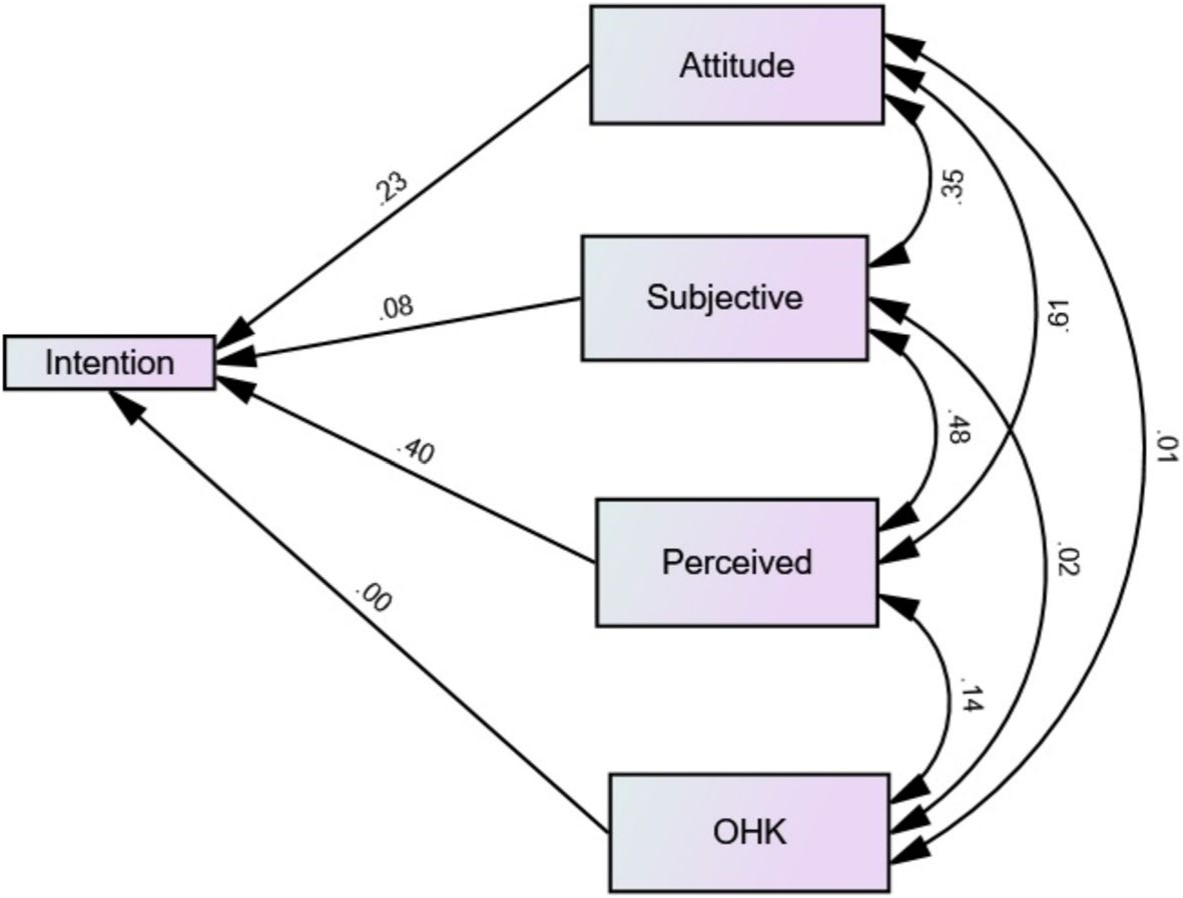
Structure equation modeling analysis. Single-headed arrows indicate the hypothesized direction of causality, and double-headed arrows indicate nondirectional associations. Numbers adjacent to arrows represent the standardized direct effect

The model fitted well with χ2 = 0.55 (*p* = 0.568), TLI = 2.89, MSE = 0.0001, GFI = 0.745. Each relationship in the research model and the variance explained (*R*^2^ value) by each relationship are shown in Fig. 2. The model was controlled for all confounding variables such as sex. The intention to improve oral health behaviors was significantly and positively predicted by attitude (β = 0.299, *p* < 0.0001), subjective norms (β = 0.035, *p* < 0.0001), and perceived behavioral control (β = 0.144, *p* < 0.0001). All measure significantly predicts the intention except for oral health knowledge (*p* = 0.890) (Table 6). This indicates that the higher the attitude, the higher the positive social pressure and the higher perceived behavioral control were significantly associated with the higher OHBI. In addition, attitude was the strongest predictor of OHBI. Together, these variables explained 72% of the variance in OHBI.

**Table 6:** Structural equation model of the study hypotheses

			Beta- coefficient	SE	p
Intention	←	Attitude	0.229	0.027	***
Intention	←	Subjective Norms	0.035	0.011	***
Intention	←	Perceived Behavior Control	0.144	0.011	***
Intention	←	Oral Health Knowledge	0.008	0.056	0.890

## Discussion

This study used a structural equation model of the TPB to determine the predictors of the intention to improve oral health behavior in the adult Saudi population. The variables of the TPB model explained 72% of the variance in OHBI, which indicates a good model fit. This reveals that the TPB has high predictive value for OHBI, which is in line with previous studies that found that the TPB accounted for comparable percentages of the variance (64–66%) in oral health behaviors [24, 31, 32]. However, other studies showed that the TPB explained a lower percentage of variance (39–50%) in behavioral intentions [24, 33]. This difference might be due to the difficulty in the process from behavioral intention to actual behavior. In addition, it could be due to the use of regression analysis instead of SEM, leading to regression bias and measurement error.

Attitudes, subjective norms, and perceived behavioral control were significant predictors of OHBI. This finding is similar to those of studies performed in other countries, such as Ireland, Australia and Ethiopia [25, 27, 31, 33, 34]. Interestingly, other studies found that subjective norms did not affect OHBI [24, 29, 30]. Shitu et al. suggested that these contradictions could be due to sociodemographic differences in the study populations [31]. In addition, in North America, people tend to be more individualistic and have fewer family bonds compared to African and Arab communities, which may weaken the influence of subjective norms. Of the TPB constructs, attitude was the strongest predictor of OHBI. This implies that participants who had favorable attitudes toward oral health behavior had a stronger intention of improving their behavior. Attitude, which was derived from Azjen’s theory of planned behavior, has consistently showed the strongest effects on behavioral intention [21]. Individuals may thus consider the outcomes before engaging in a given behavior; in other words, people rely on experience to determine their intention to improve their oral health behavior. This finding is in line with those of Shitu et al. [31], Dumitrescu et al. [24], and Buunk-Werkhoven et al. [27].

In general, the greater the perceived self-control is and the stronger the subjective norm, the stronger an individual’s intention to engage in a given behavior [21]. When applying the TPB, we found that the participants’ perceived behavioral control significantly predicted their OHBI. This finding confirms higher self-efficacy is associated with better OHB and is in accordance with previous studies conducted on flossing and attendance at dental clinics [29, 30]. Of the TPB variables, subjective norms were the weakest predictor of OHBI, indicating that participants were not influenced by their close relatives. A possible explanation is that our participants were independent adults who made their own decisions. In contrast to our results, other studies conducted in Indonesia and Iran found that subjective norms had a stronger effect on OHBI than attitude [33, 35]. This inconsistency may be due to variations in sociodemographic characteristics among the study subjects. For instance, Bramantoro et al.’s study found that subjective norms were a significant factor for 12–16-year-old students indicating that they were influenced by their teachers and parents and still possessed a sense of social relatedness [33]. Saudi Arabian adults tend to have a high level of individualization in their lifestyle.

Usually, one would assume that the more accurate people’s OHK is, the more likely they would be to improve their OHB. Surprisingly, we found that OHK was not a predictor of OHBI, even though OHK scores were high. This finding contrasts with those of previous studies that found that OHK had a strong influence on OHBI [24, 27]. Indeed, Dumitrescu et al. claimed that OHK was linked to attitude, in that greater OHK led to stronger attitudes and thus indirectly affected OHBI [24]. According to social learning theory, knowledge alone cannot change health behavior and intention. Studies suggest that education and knowledge only temporarily impact human behaviors and intentions [36, 37].

This study had limitations that should be addressed in future research. First, despite our large sample size, a large proportion of the study participants were female (86%) and 20–30 years old (61%), which may have biased the results and limited the generalizability of the study findings. Therefore, our results might not be representative of the wider population of Saudi Arabia. Second, the use of online surveys and social media might bias the results since participants have access to online information regarding oral health. Third, we used a snowball sampling strategy which might cause selection bias. Fourth, because of the cross-sectional nature of the study, it cannot demonstrate the cause-effect relationship. Finally, as the TPB is a behavioral model, we did not consider other factors, such as demographic and environmental factors. Future studies should consider samples with more variation to ensure the generalizability of the findings to the total population of Saudi Arabia. For example, other venues in addition to malls should be considered in future studies to facilitate enrollment of men. In addition, future studies should explore the Reasoned Action Approach, which is an extension of the TPB that divides the theory constructs into subcomponents and measures behavioral intention within human action.

## Implication

The results of this study emphasize the importance of the TPB constructs for predicting OHBI. These constructs establish a foundation for organizations that design oral health promotion and intervention programs. The findings indicate the importance of focusing on attitudinal changes and encouraging high levels of self-control and self-efficacy in interventions to improve OHB. Effective and efficient programs for oral health promotion and intervention at the individual and community levels should be organized, not only for children and adolescents but also adults and significant others. For example, dental professionals and students should mobilize and reinforce oral health promotion at the local and regional levels. In order to promote good self-oral health, such as using fluoridated toothpaste and flossing daily, these health promotion programs should be conducted in clinics, social and cultural settings. Practical hands-on activities should be included in such programs to help acquire correct oral health behaviors. In addition, providers should be encouraged to ensure that oral care products, such as toothbrushes and fluoridated toothpaste, are available and inexpensive.

## Conclusions

Regardless of the limitations of this study, the findings support the validity of the TPB in predicting the intention to improve OHB in adults, especially young females. Attitudes, subjective norms, and perceived behavioral control were significant predictors of intentions to improve oral health behaviors. The best way to improve OHBI is to change the attitudes, enhance perceived behavior control and emphasize the positive influence of close relationships. Following these strategies will improve the adherence to the recommended practices.

## Data Availability

The datasets used and/or analysed during the current study available from the corresponding author on reasonable request.

## Abbreviations

A: Attitude
OHK: oral health knowledge
OHB: oral health behavior
OHBI: oral health behavioral intentions
PBC: perceived behavioral control
SEM: structural equation modeling
SN: Subjective norm
TPB: theory of planned behavior
